# Targeted genomic profiling identifies frequent deleterious mutations in *FAT4* and *TP53* genes in HBV-associated hepatocellular carcinoma

**DOI:** 10.1186/s12885-019-6002-9

**Published:** 2019-08-08

**Authors:** Fung-Yu Huang, Danny Ka-Ho Wong, Vivien Wai-Man Tsui, Wai-Kay Seto, Lung-Yi Mak, Tan-To Cheung, Keane K.-Y. Lai, Man-Fung Yuen

**Affiliations:** 1 Department of Medicine, The University of Hong Kong, Queen Mary Hospital, Hong Kong SAR, Hong Kong; 2 State Key Laboratory of Liver Research, The University of Hong Kong, Hong Kong SAR, Hong Kong; 3 Department of Surgery, The University of Hong Kong, Queen Mary Hospital, Hong Kong SAR, Hong Kong; 4 Depatment of Pathology, City of Hope National Medical Center, Duarte, CA USA; 5 Department of Molecular Medicine, Beckman Research Institute of City of Hope, Duarte, CA USA; 6 City of Hope Comprehensive Cancer Center, Duarte, CA USA

**Keywords:** Hepatocellular carcinoma, Targeted sequencing, Deleterious mutations, Customized therapies, Gene silencing

## Abstract

**Background:**

Hepatitis B virus (HBV) is the major risk factor for hepatocellular carcinoma (HCC). The molecular mechanisms underlying HBV-associated HCC pathogenesis is still unclear. Genetic alterations in cancer-related genes have been linked to many human cancers. Here, we aimed to explore genetic alterations in selected cancer-related genes in patients with HBV-associated HCC.

**Methods:**

Targeted sequencing was used to analyze six cancer-related genes (*PIK3CA, TP53, FAT4, IRF2, HNF4α* and *ARID1A*) in eight pairs of HBV-associated HCC tumors and their adjacent non-tumor tissues. Sanger sequencing, quantitative PCR, Western-blotting and RNAi-mediated gene knockdown were used to further validate findings.

**Results:**

Targeted sequencing revealed thirteen non-synonymous mutations, of which 9 (69%) were found in *FAT4* and 4 (31%) were found in *TP53* genes. Non-synonymous mutations were not found in *PIK3CA*, *IRF2*, *HNF4α* and *ARID1A*. Among these 13 non-synonymous mutations, 12 (8 in *FAT4* and 4 in *TP53*) were predicted to have deleterious effect on protein function by in silico analysis. For *TP53,* Y220S, R249S and P250R non-synonymous mutations were solely identified in tumor tissues. Further expression profiling of *FAT4* and *TP53* on twenty-eight pairs of HCC tumor and non-tumor tissues confirmed significant downregulation of both genes in HCC tumors compared with their non-tumor counterparts (*P* < 0.001 and *P* < 0.01, respectively). Functional analysis using RNAi-mediated knockdown of *FAT4* revealed an increased cancer cell growth and proliferation, suggesting the putative tumor suppressor role of *FAT4* in HCC.

**Conclusions:**

This study highlights the importance of *FAT4* and *TP53* in HCC pathogenesis and identifies new genetic variants that may have potentials for development of precise therapy for HCC.

**Electronic supplementary material:**

The online version of this article (10.1186/s12885-019-6002-9) contains supplementary material, which is available to authorized users.

## Background

Hepatocellular carcinoma (HCC) is one of the most common malignant tumors worldwide. With an incidence of over 700,000 new cases per year, it ranks the sixth most common cancer and the third leading cause of cancer-related deaths worldwide [1]. China alone accounts for about 50% of the total number of cases and deaths [2]. Most cases of HCC are associated with chronic infection of hepatitis B virus (HBV) and/or hepatitis C virus (HCV). Other factors such as alcohol consumption, smoking, aflatoxin B exposure, diabetes, obesity, and non-alcoholic fatty liver disease (NAFLD) may act either as amplifiers of the effects of viral hepatitis or as independent risk factors of HCC [3]. Although there are advances in HCC diagnosis and treatment in recent decades, most HCC are still asymptomatic until at a late stage and hence resulting in a poor long-term prognosis and with limited therapeutic modalities [4]. Therefore, it is necessary to identify genomic alterations underlying the pathogenesis of HCC to pinpoint efficient therapeutic targets for early diagnosis and treatment of this deadly disease, as well as to improve its prognosis in affected patients [5].

Accumulation of genetic alterations in oncogenes, tumor-suppressor genes, cell adhesion molecules and DNA repair genes are characteristic features of many human cancers including HCC [6]. Over the past few years, next-generation sequencing (NGS) has profoundly advanced our understanding of cancer genomics. The identification of disease driver genes in some solid tumors holds promise for precision medicine, such as *ALK* inhibitors in non-small cell lung cancer with an *ALK* rearrangement or *BRAF* inhibitors in melanoma with a *BRAF* mutation [7, 8]. Unfortunately, liver cancer has not yet reached the point of molecular-based treatment stratification, mainly due to incomplete understanding of the molecular landscape of HCC in particular the genomic alterations caused by different etiological factors [9]. Systematic efforts to elucidate the comprehensive somatic changes in a large group of viral-associated (both HBV and HCV) HCC tumor samples with an international contribution efforts are still underway (http://cancergenome.nih.gov/).

Although the genomic alterations underlying HCC have not been clearly understood, a broad variety of pathways activated in HCC have been reported including the Wnt/β-catenin, p53/cell cycle, chromatin remodeling complex, PI3K/Ras, and oxidative stress signaling [10]. Genetic alterations identified in key genes involved in these pathways generally present with different frequency in different cancer types and etiology background [10, 11]. For example, the incidence of mutation in the well-known tumor suppressor gene *TP53* varied from 5 to 70% depending on cancer types and stage [12]. In HCC, the rates of *TP53* mutation varied significantly between African or Asian (10–60%) and Western countries (10–20%) [13]. Presence of *PIK3CA* mutation has been controversial with approximately 35.6% of HCC cases in Korea [14], 28% in Italy [15] and 0% in Japan [16]. By using NGS technologies, somatic mutations in several novel cancer-related genes such as *ARID1A* (7.53%), *HNF4α* (0.88%)*, FAT4* (4.71%) and *IRF2* (1.06%) have been identified and suggested to be associated with HCC [17]. However, these studies were performed in patients with HCC of heterogeneous etiologies, and the role of genetic changes in these genes in the development of HBV-associated HCC is largely unknown.

To explore whether genetic changes in cancer-related genes can be identified in chronic hepatitis B patients with HCC, we performed targeted sequencing to detect the incidence of mutations in six selected cancer-related genes including *ARID1A, TP53, FAT4, HNF4α, PIK3CA* and *IRF2*. These genes have been suggested to play functional roles in chromatin remodeling (*ARID1A*), tumor suppression (*TP53* and *FAT4*), transcription activation (*HNF4α* and *IRF2*), and oncogenic development (*PIK3CA)* (Additional file 1: Table S1) [18–20]. Identification of the key genes and the related mechanisms could provide a better understanding on HCC pathogenesis and develop effective therapeutic strategies. Hence, we aimed to identify genetic changes in cancer-related genes in HBV-associated HCC and explore whether they play roles in the process of HCC pathogenesis.

## Methods

### Sample preparation and nucleic acids extraction

Eight pairs of tumor and their adjacent non-tumor tissues were collected from Asian patients who had HBV-related HCC and had undergone surgical resection at the Queen Mary Hospital, Hong Kong. Patients with other risk factors, such as HCV infection, heavy alcohol consumption, nonalcoholic steatohepatitis (NASH) and smoking were excluded in this study. These tissues were rapidly snap-frozen in liquid nitrogen and stored at -80 °C freezers for future analysis. Written informed consent was obtained from all patients. This study was approved by the Institutional Review Board (UW 17–312), University of Hong Kong. Nucleic acids were extracted from about 30 mg of liver tissues by the QIAamp Allprep Kit (Qiagen, Hilden, Germany), according to the manufacturer’s instructions. This extraction kit allows simultaneous extraction of DNA, RNA, and protein from the same piece of liver tissue. During RNA isolation, on-column DNase digestion was performed using RNase-free DNase (Qiagen) to get rid of DNA contamination. The quantity and quality of the nucleic acids were determined by using the NanoDrop and the Qubit fluorometer (Thermo Fisher Scientific, MA, USA). For the validation of gene expression level, RNA extracted from additional 20 pairs of tumor and non-tumor tissues from HBV-associated HCC patients were used.

### Library preparation and targeted sequencing

Briefly, 100 ng of DNA from tumor or non-tumor tissues was fragmented with a Covaris M220 instrument (Covaris, Woburn, USA). Library preparation and custom target enrichment were performed with the KAPA Library Preparation kit for Illumina platforms (Kapa Biosystems, Wilmington, USA) and NimbleGen SeqCap EZ Library kit (Roche, Madison, WI, USA), respectively, following the manufacturer’s protocol. The captured library was then amplified and sequenced using HiSeq 2000 (Illumina, San Diego, USA). Library preparation and targeted sequencing were performed by Centre for Genomic Sciences, The University of Hong Kong.

### Targeted sequencing data analysis

The base calling and sequence alignment were performed using the Illumina pipeline (version 1.4) with default parameters [21]. The sequence reads were mapped to the reference human genome (hg19) using Burrow Wheeler Aligner (BWA) version 0.6.2 [22]. The optimization of sequence alignment, variant calling and annotation were performed using Genome Analysis Toolkit (GATK) version 3.2 [23]. The effects of missense variants and amino acid substitutions on protein function were predicted with four algorithms [SIFT [24], Polyphen2 [25], Mutation Taster [26] and LTR [27]].

### Mutation verification by sanger sequencing

All the significant non-synonymous mutations were validated by Sanger sequencing. Primer pairs were designed to amplify the target sites using Primer 3 software (http://bioinfo.ut.ee/primer3/) (Additional file 2: Table S2). Purified PCR products containing the potential variants were sequenced using the ABI 3730 DNA Analyzer (Applied Biosystems, Foster City, CA) to further ascertain the precision of the variants identified by targeted sequencing.

### Cell culture

The human liver cancer cell lines (SNU-387, Huh7, HepG2, HepG2.2.15 and Hep3B) were obtained from the American Type Culture Collection (Manassas, VA, USA). Normal liver cell line, L02 was obtained from the Shanghai Institutes for Biological Sciences, and Chinese Academy of Sciences. All the cell lines were kept within 10 passages and have been tested for mycoplasma contamination using PCR method [28]. Cells were maintained in RPMI-1640 medium with 10% fetal bovine serum (Thermo Fisher Scientific) in a humidified incubator with 5% CO_2_ at 37 °C.

### siRNA knockdown of FAT4

Transfection was performed with Lipofectamine 3000 reagent (Invitrogen) following the manufacturer’s protocol. Briefly, SNU-387 cells were seeded in plates one day before transfection to ensure suitable cell confluency on the day of transfection. Ambion® Silence® select pre-designed siRNAs targeted *FAT4* (Invitrogen) were used at a final concentration of 50 nM siRNA with non-specific sequences were used as controls. Cells were harvested at day 2 post-transfection, or as indicated.

### Cell growth and proliferation analysis

SNU-387 cells were cultured in 12-well plate at about 5 × 10^4^ per well for cell growth assay and in 96-well plates at about 5 × 10^3^ per well for cell proliferation assay. Cells were transfected with siRNA targeting *FAT4* or control siRNA for 24, 48, 72 h. Cells were observed under the phase contrast microscopy for changes in morphology and cell numbers at the designated time. For cell growth analysis, cells were trypsinized and diluted 1:1 with 0.4% trypan blue (sigma) and viable cells were counted with a hemocytometer (Sigma). For cell proliferation assay, 10 μl of Cell Counting Kit-8 solution was added into each well containing 100 μl culture medium and incubated for 2 h at 37 °C. The optical density value of each well was measured by absorbance at 450 nm in a microplate reader. Experiments were performed in duplicates.

### Real-time PCR analysis of gene expression

RNA was extracted from liver cell lines using TRIzol reagent (Thermo Fisher Scientific), following the manufacturer’s protocol. RNA concentrations and integrity were determined using the NanoDrop 2000 Spectrophotometer (Thermo Fisher Scientific). Gene expression was measured by qRT-PCR using SYBR Green PCR master mix (Bio-Rad, Herculus, CA). Gene expression levels were normalized with GAPDH as an internal control gene and with adjacent non-tumor samples using the 2^-∆ΔCT^ method. Primer sequences used for gene amplification are listed in Additional file 2: Table S2.

### Western blot analysis

Protein extraction from cultured cells was performed using the Mammalian Cell Lysis Reagent (Thermo Fisher Scientific). Protein concentration was determined by Bradford protein assay (Thermo Fisher Scientific), following the manufacturer’s protocol. Equal amounts of total protein were loaded on 7% Tris-acetate polyacrylamide gels, transferred to a PVDF membrane, blocked with 5% milk, and then probed with relevant primary antibodies to FAT4 and p53 (Santa Cruz, CA), α-tubulin and β-actin (Cell Signaling, MA) overnight at 4 °C. Protein expression was assessed by ECL detection system (GE Healthcare, NJ) and band intensities were quantified using the Image J software (NIH, Bethesda, MD).

### Statistical analysis

Continuous variables were expressed as mean ± standard error (SEM) and analyzed using the student’s t-test. All statistical analysis was performed using GraphPad Prism 5.0 (GraphPad Software, Inc. San Diego, CA). A *P* value of less than 0.05 was considered statistically significant.

## Results

### Patient characteristics and sequencing quality

The clinical characteristics of the HBV patients with HCC are shown in Additional file 3: Table S3. Genetic alterations in coding regions and selected regulatory and intronic regions in the six cancer-related genes (Additional file 1: Table S1) were successfully sequenced using targeted sequencing method. After filtering reads with low sequence quality or sequencing adaptor, we obtained a total of > 1.0 Mb high-quality reads per sample (Additional file 4: Table S4). More than 99.9% of the yielded clean reads could be uniquely mapped to the human reference genome *hg*19, achieving 600x on target mean coverage in all the 16 samples. This high coverage (> 98.6%) of targeted regions (≥8x) allow a highly reliable detection of all variations in targeted regions (Additional file 4: Table S4).

### Mutation identification

**Fig. 1:**
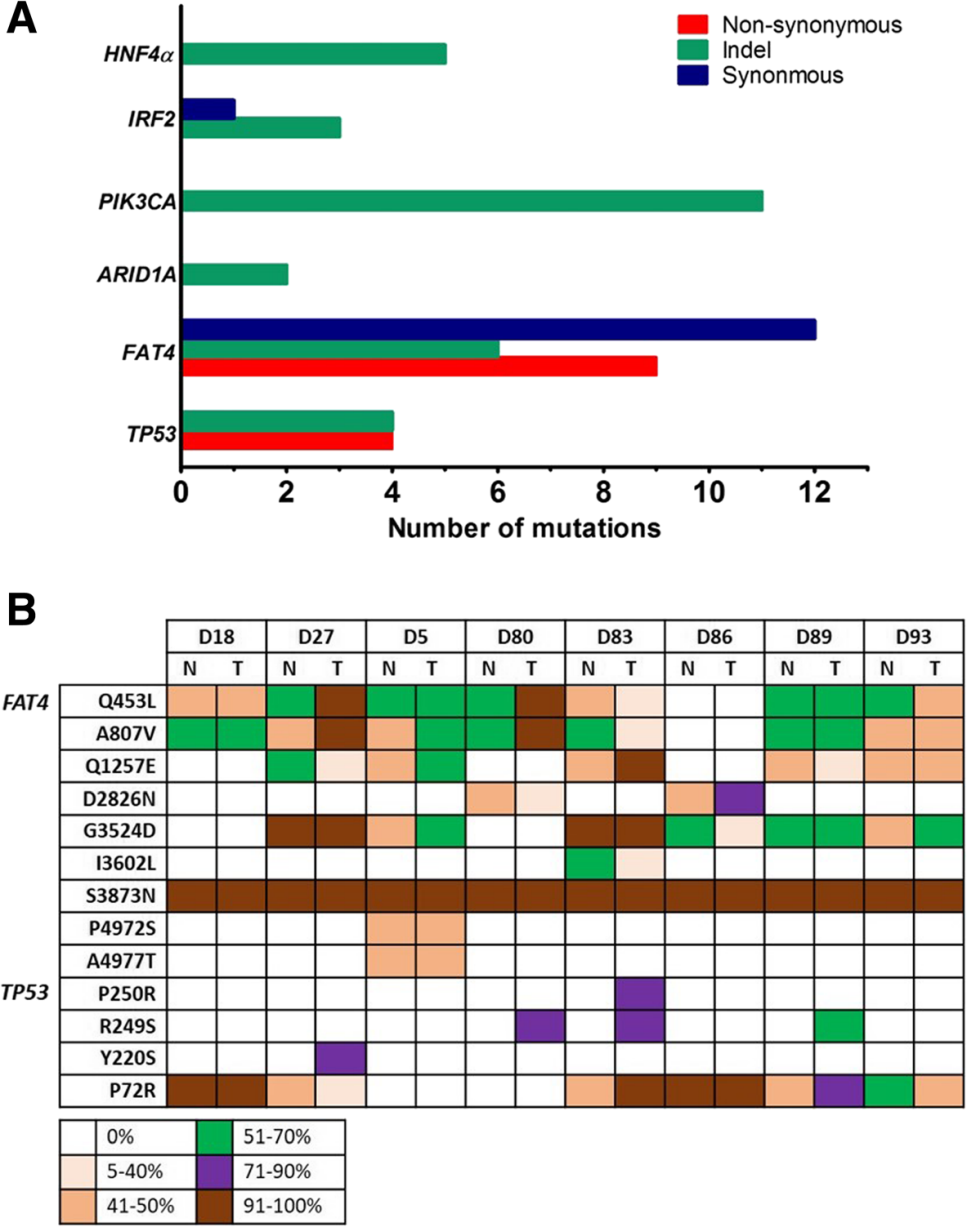
Overview of the number and frequency of the mutations. (**a**) Bar plot indicating the number and type of mutations detected in the six cancer-related genes. (**b**) Summary list of the frequency of non-synonymous mutation result in coding change detected in the study cohorts. Top row indicated the tissue samples. Left-handed row indicated the mutation changes detected in *FAT4* and *TP53* genes. The color-coded legend indicated the variant frequency detected in each sample. (T: tumor tissue; N: adjacent non-tumor tissue)

**Fig. 2:**
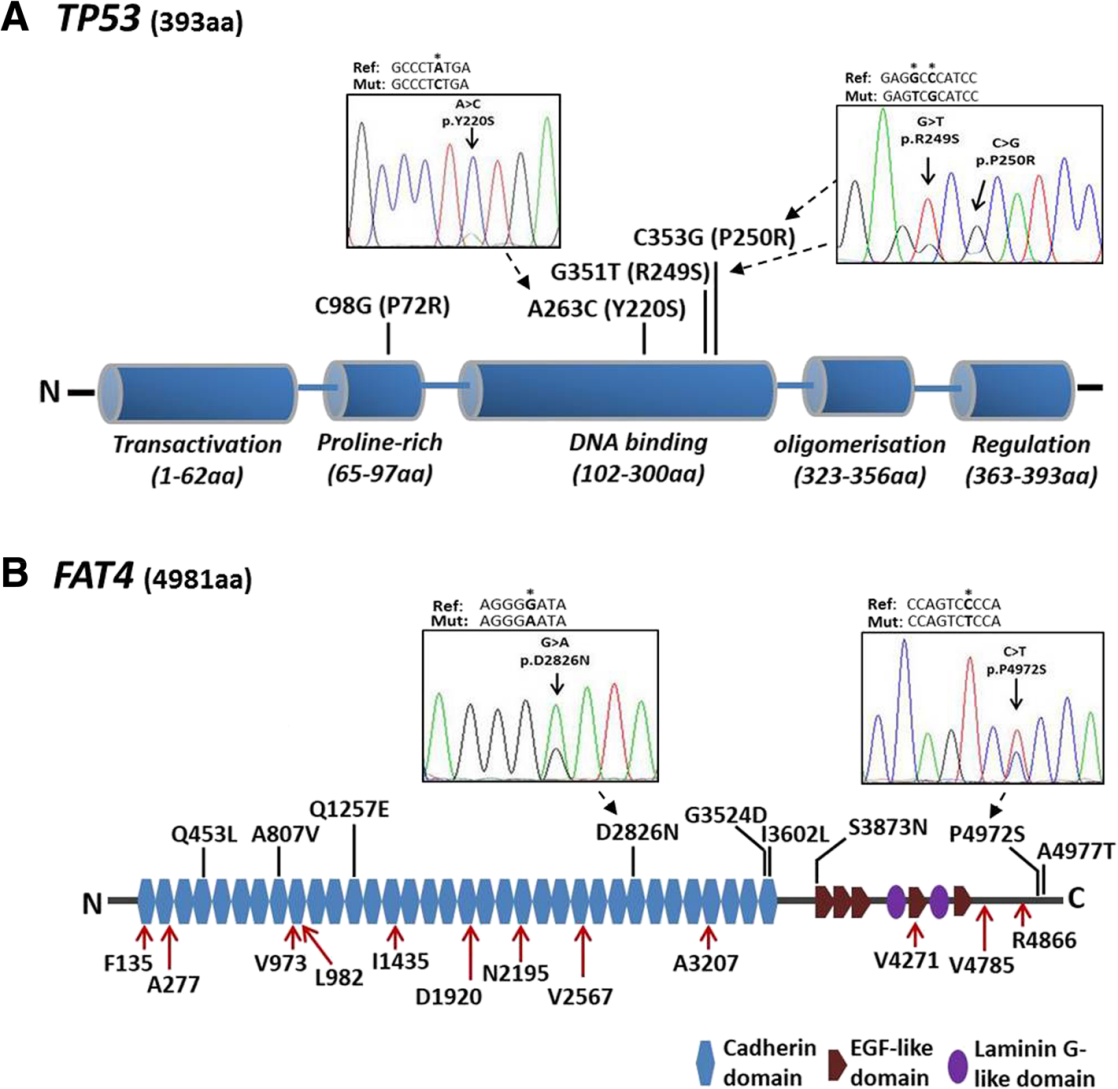
Graphic diagram showing the protein domains and distribution of significant genetic mutations identified in *TP53* and *FAT4.* (**b**) p53 protein with 393 amino acids and consists of transactivation, proline-rich, DNA binding, oligomerization and regulation domains. Partial electropherograms of three representative mutations resulted in amino acid alterations (Y220S, R249S and P250R) identified in *TP53* are shown. (**b**) *FAT4* protein with 4981 amino acids and consists of 34 cadherin domains, 5 EGF-like domains and 2 laminin G-like domains. Partial electropherograms showing two representative non-synonymous mutations (P4972S and D2826N) identified in *FAT4*. The locations of the genetic variants are indicated by red arrows for synonymous mutation and black bars for non-synonymous mutation. The position of mutation is bolded and marked with asterisk in reference sequence for *TP53* (NM_001125115) and *FAT4* (NM_024582). (Ref: reference sequence; Mut: mutated sequence)

Genetic variants with high-quality allelic frequency ≥ 5% were identified in all the samples (Figs. 1 and 2, and Additional files 5, 6, 7 : Table S5–7). A total of 57 somatic mutations were identified. Of these, 13 (22.8%) were non-synonymous somatic mutations, 31 (54.4%) were insertions/deletions (indels) and 13 (22.8%) were synonymous mutations (Fig. 1 and Additional files 5, 6, 7: Table S5–7). 70% (40/57) of these mutations were indexed in the single nucleotide polymorphism database (dsSNP) and 75% (9/13) of the non-synonymous mutations were listed in the Catalogue Of Somatic Mutations In Cancer (COSMIC) database. The most commonly mutated genes were *FAT4* (27/57, 47.4%), *PIK3CA* (11/57, 19.3%), *TP53* (8/57, 14%), *HNF4α* (5/57, 8.8%), *IRF2* (4/57, 7%), and *ARID1A* (2/57, 3.5%) (Fig. 1a).

### Non-synonymous mutations

Thirteen non-synonymous mutations were identified, of which 9 (69%) were found in *FAT4* and 4 (31%) were found in *TP53* (Figs. 1 and 2). Non-synonymous mutations were not in *PIK3CA*, *IRF2*, *HNF4α* and *ARID1A*. Further in silico analysis using different software [SIFT [24], PolyPhen2 [25], Mutation Taster [26] and LRT [27]] predicted that 12 of the 13 non-synonymous mutations might cause significant changes in protein structure and hence were potentially deleterious or damaging on protein function (Additional file 5: Table S5). Of the 9 non-synonymous mutations identified in *FAT4*, 8 (except S3873 N) were predicted to be deleterious on protein function (Additional file 5: Table S5). Six of these non-synonymous mutations were located in the cadherin domains (6/9, 66.7%) and annotated in the COSMIC database, while the other three were located in the C-terminal ends (3/9, 33.3%) (Fig. 2b). The remaining four non-synonymous mutations were present in *TP53* gene (4/13, 31%) (Fig. 1). P72R is located in the proline-rich region, and the other three mutations Y220S, R249S and P250R are localized in the DNA binding domain (Fig. 2a). These latter 3 mutations were predicted to be “deleterious” by all the four prediction algorithms, and were only detected in tumor tissues but not in their adjacent non-tumor controls (Fig. 1B and Additional file 5: Table S5). Notably, the hot spot R249S mutation was detected in 3/8 (37.5%) patients. Additionally, some genetic variants in *FAT4* and *TP53* were present in both tumor and their adjacent non-tumor counterparts with tumor tissues generally harboring a higher allelic frequency of mutations (Fig. 1b).

### Synonymous mutations and indels

There were 13 synonymous mutations identified in the exonic regions in *FAT4* (12/13, 92.3%) and *IRF2* (1/13, 7.7%) genes (Additional file 6: Table S6). A total of 31 indels were found in upstream, intronic and 3′ or 5’untranslated regions (UTRs) (Fig. 1a and Additional file 7: Table S7) of these 6 targeted genes. *PIK3CA* was detected with the highest frequency of indel mutations (11/31, 35.5%), followed by *FAT4* (6/31, 19.4%), *HNF4α* (5/31, 16.1%), *TP5*3 (4/31, 12.9%), *IRF2* (3/31, 9.7%) and *ARID1A* (2/31, 6.5%) (Additional file 7: Table S7).

### Confirmation of the identified variants by sanger sequencing

Sanger sequencing was performed to confirm the accuracy of the 13 non-synonymous genetic variants in *FAT4* and *TP53* genes, with 12 of them were predicted to have disease-causing potential in samples identified by targeted sequencing (Fig. 1b). All these genetic variants presented in the same sample identified by targeted sequencing could be validated by Sanger sequencing (Fig. 2 and Additional file 8: Figure S1). The results showed complete consistency between the two methods, suggesting that the targeted sequencing method used in this study provides high accuracy and with no false-positive rate.

### Downregulation of FAT4 and TP53 in liver tumor tissues and cell-lines

**Fig. 3:**
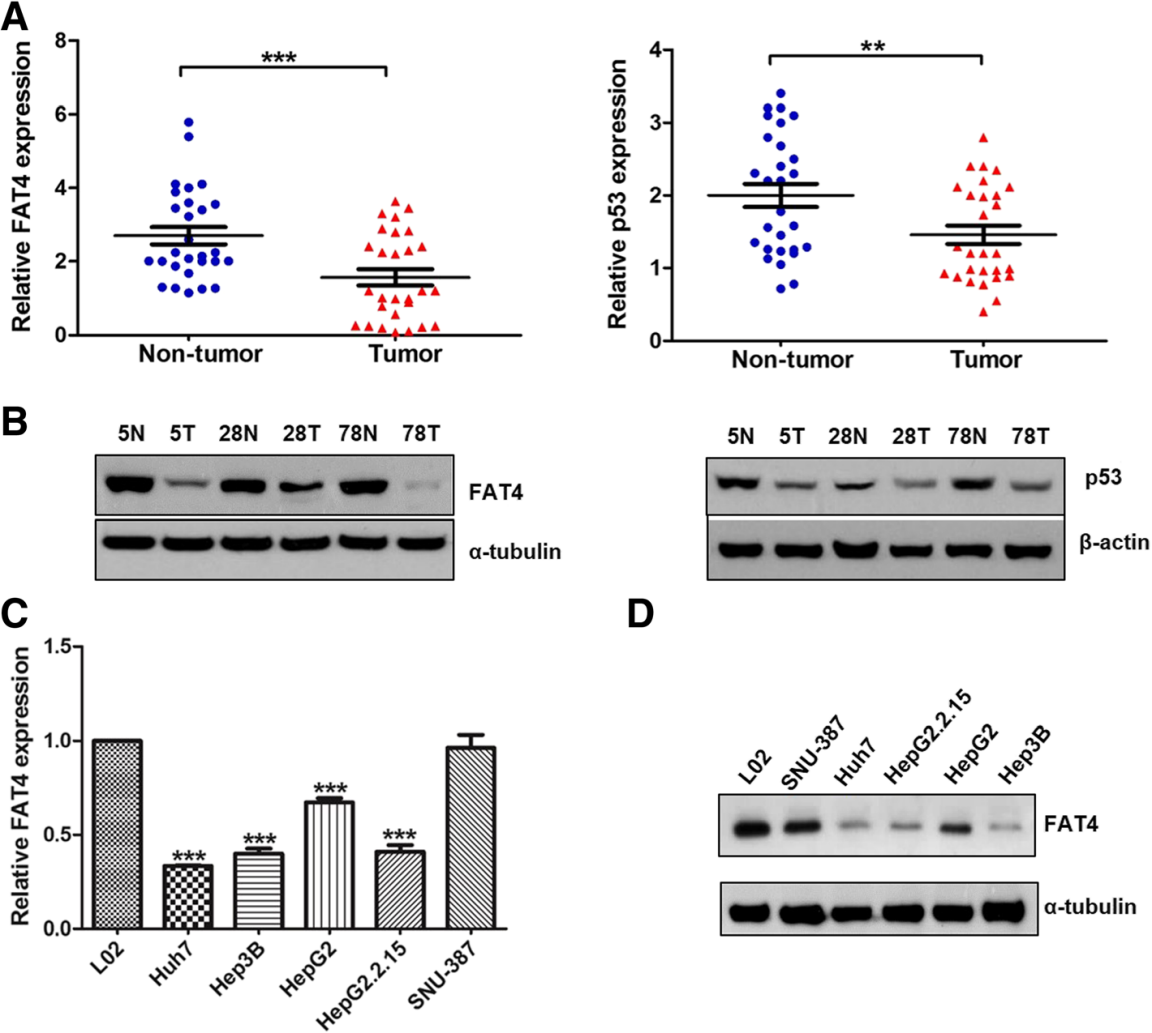
The mRNA and protein expression of *FAT4* and p53 in the human liver tissues and cell-lines. (**a**-**b**) Downregulation of mRNA and protein expression in *FAT4* and p53 in the tumor tissues compared with their adjacent non-tumor controls. (**c**) The mRNA expression of *FAT4* was significantly downregulated in four liver cancer cell-lines. (**d**) The protein expression levels of *FAT4* in the cell-lines. Data represent means ± SEM from three independent experiments (**P < 0.01, ***P < 0.001)

To further examine the biological significance of *FAT4* and *TP53* in HCC, we studied *FAT4* and *TP53* expression in a total of 28 pairs of tumor and non-tumor tissues and in six liver cell lines. As shown in Fig. 3a, the mRNA expression levels of *FAT4* and *TP53* were significantly downregulated in tumor tissues compared with their adjacent non-tumor counterparts (*P* < 0.001 and *P* < 0.01, respectively). Western blot analysis also revealed that *FAT4* and p53 protein levels were lower in tumor tissues compared with the corresponding non-tumor tissues (Fig. 3b). The *FAT4* mRNA expression levels were also significantly reduced in the four liver cancer cell-lines (Hep3B, HepG2, HepG2.2.15 and Huh7) compared with the normal cell-line L02 (*P* < 0.001) (Fig. 3c). Similar results were obtained by Western blot analysis of *FAT4* protein expression (Fig. 3d). These data suggest that *FAT4* is repressed during hepatocarcinogenesis.

### siRNA knockdown of FAT4 promotes cell growth and proliferation

**Fig. 4:**
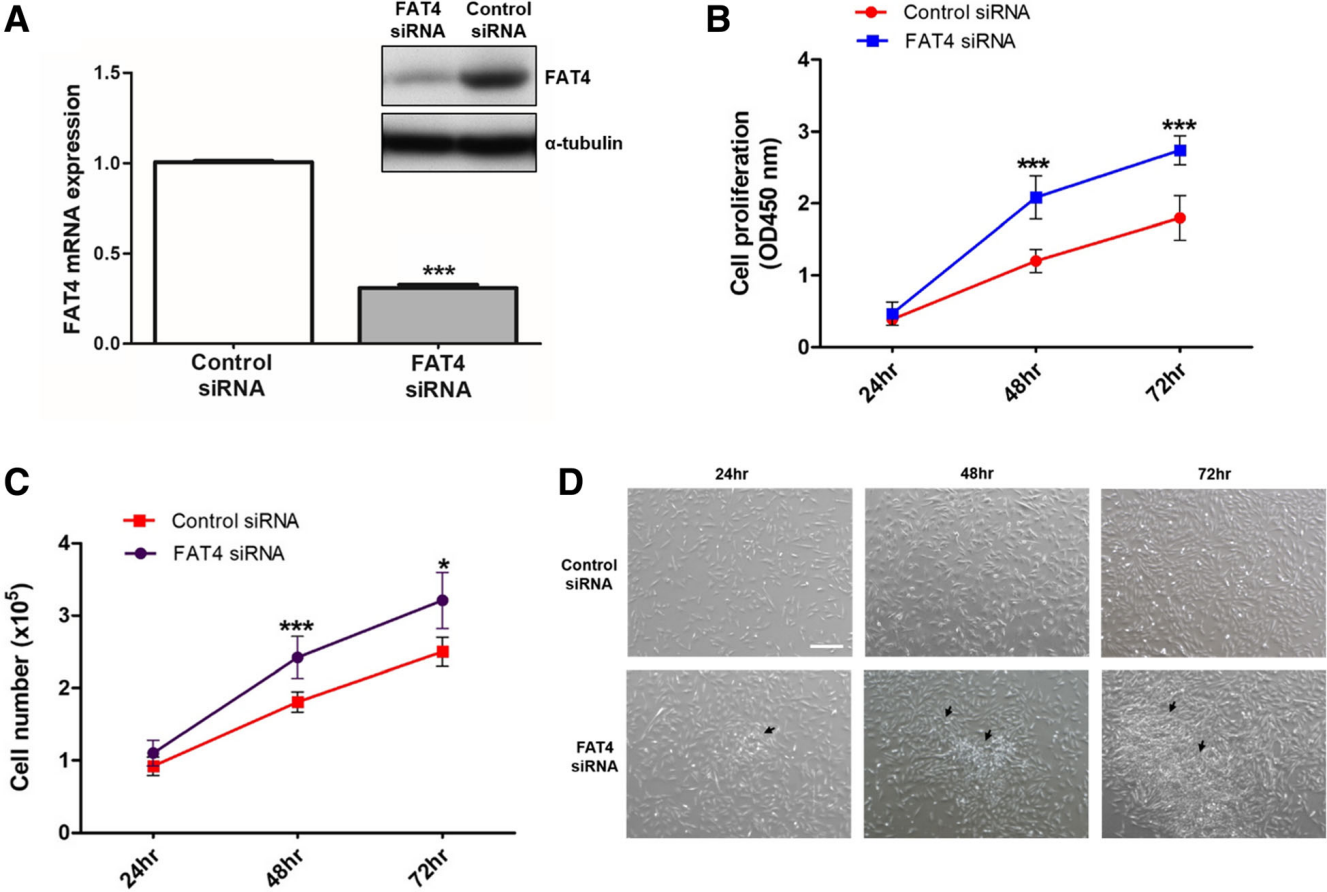
Effects of *FAT4* silencing on liver cancer cell growth and proliferation. (**a**) Confirmation on the efficiency of *FAT4* silencing by assessing the mRNA and protein expression levels after siRNA transfection at 24 h and 48 h, respectively. (**b**) siRNA knockdown of *FAT4* significantly increased cell proliferation compared with cells transfected with control siRNA at 48 and 72 h. (**c**) Significantly increased in cell growth was observed in cells transfected with *FAT4* siRNA at 48 and 72 h after transfection. (**d**) Representative morphological appearance of SNU-387 cells treated with *FAT4* siRNA by phase contrast microscopy at 24, 48, 72 h (40× magnification). Arrows indicate rapidly proliferating cell clumps. Data are mean values ± SEM of triplicate determinations (*P < 0.01 and ***P < 0.0001)

The tumor suppressor role of *TP53* is well characterized in HCC but not for *FAT4*. We next explored the functional role of *FAT4* in liver cancer cells using siRNA-mediated knockdown of *FAT4* expression. HBV-associated HCC cell-line, SNU-387 was chosen for knockdown experiment as the expression of *FAT4* in SNU-387 cells was higher than the other 4 cancer cell-lines which makes it a better candidate to study the effect of *FAT4* knockdown. The efficiency of siRNA-mediated *FAT4* knockdown was confirmed by reduction in both mRNA and protein levels compared with cells transfected with control siRNA (both with *P* < 0.0001) (Fig. 4a). We next studied the effect of *FAT4* knockdown on cell growth and proliferation. As shown in Fig. 4b, cell proliferation was significantly enhanced after 48 and 72 h in cells with *FAT4* siRNA transfection compared with cells transfected with control siRNA (both with *P* < 0.0001). Similarly, cell growth was significantly increased by 33 and 24% in cells transfected with *FAT4* siRNA at 48 h (*P* < 0.0001) and 72 h (*P* < 0.01) compared with cells transfected with control siRNA, respectively (Fig. 4c). Morphologic changes were observed with cells showing rapidly growth without contact inhibition and forming clonal populations in *FAT4* siRNA transfected cells but not in control siRNA transfected cells at all the three time points (Fig. 4d). Taken together, knockdown of *FAT4* promotes cell growth and proliferation indicating the putative tumor suppressor role of *FAT4* in HCC.

## Discussion

In this study, we applied targeted sequencing to screen for genetic variants in HBV-related HCC samples. As expected, genetic variants were identified in all the six cancer-related genes. We identified several previously established, high-likelihood genetic variants, with either known or unknown biological significance. We focused on *FAT4* and *TP53* genes as both showed frequent non-synonymous mutations in our targeted sequencing cohort. Our in silico analysis also predicted that most of these mutations were likely to have deleterious effects on protein function, implying their involvement in HCC development in chronic hepatitis B disease.

*FAT4* belongs to the cadherin gene superfamily and encodes transmembrane proteins homologous to tumor suppressor *fat* in Drosophila [29]. The highest synonymous and non-synonymous mutations found in *FAT4* suggested its likely involvement in HCC carcinogenesis process. Non-synonymous mutations that result in amino acid coding change in *FAT4* have been reported in several cancers including colon, gastric, esophageal and liver cancers [18, 30–32]. In a study investigating somatic mutations in an individual patient with multifocal HCC, Shi et al. has also identified consistent *FAT4* mutations in different tumor loci within the same patient [31]. In our study, six non-synonymous mutations identified in *FAT4* with deleterious effects on protein function were already annotated in the COSMIC database, indicating the importance of these 6 somatic mutations in cancer development. The P4972S mutation identified in this study, although not annotated in the COSMIC database, has been predicted to influence an exonic splicing enhancer or silencer and result in disequilibrium for different isoforms of *FAT4* [30]. Our study also identified a potentially novel *FAT4* mutation, A4977T, which has not been reported in HCC, and the significance of A4977T mutation on HCC development deserves further investigation.

Unlike non-synonymous mutations, synonymous mutations change the sequence of a gene without altering the sequence of the coded protein thus are generally termed as silent mutations. However, the prevalent view on synonymous mutations are silent is changing with recent evidence indicated that synonymous mutations frequently alter exonic splicing motifs and affect mRNA splicing [33]. Moreover, genome-wide association studies (GWAS) on genetic variants and disease has revealed a substantial contribution of synonymous SNPs to human disease risk and other complex traits [34]. This implies the higher number of synonymous mutations identified in *FAT4* might also contribute to HCC risk. Taken together, our data reiterate the likely involvement of frequent *FAT4* mutations in HBV-associated HCC. We believe further functional characterization of both synonymous and non-synonymous mutations in *FAT4* will provide a better understanding of its biological relevance in hepatocellular carcinogenesis.

Expression and functional analysis indicated downregulation of *FAT4* in tumor tissues and loss of *FAT4* induced HCC cell growth and proliferation. These findings were consistent with previous reports suggesting the tumor suppressor role of *FAT4* in human cancers [18, 35]. However, knowledge about the exact functional role of *FAT4* in HCC and its involvement in downstream signaling activation are still scarce. Thus, further delineation of the functional role of *FAT4* as a HCC candidate gene especially using in vivo animal models are warranted.

There is a strong association between *TP53* mutations and HCC [36]. Our findings also revealed frequent non-synonymous *TP53* mutations with disease-causing effects in HCC. The P72R mutation in the proline-rich region was reported to affect the structure of the putative SH3-binding domain [37]. The presence of Y220S and R249S mutations are proven to disrupt its transactivation activity according to the International Agency for Research on Cancer (IARC) *TP53* database. Notably, we detected a high frequency of hot spot R249S mutation in tumor tissues. This finding is consistent with the reported mutation of R249S in > 30% of HCC cases in geographical areas of high HCC incidence [38]. The R249S mutation was induced by aflatoxin metabolites and this mutant *TP53* could interact with HBx leading to cell proliferation, suggesting that the R249S mutation is an early mutational event in hepatocarcinogenesis [39, 40]. Of note, the P250R is a novel genetic variant predicted to be deleterious by all four prediction algorithms and was not reported in any reference database. It resides in the DNA recognition region, in which a change in amino acid could affect the DNA binding ability of *TP53* and therefore its associated transcriptional function. Our data further emphasize the importance of *TP53* mutation in HBV-related HCC. The pathological link between genetic alterations leading to the loss of *TP53* function and the initiation and progression of HCC with different etiologies warrant further confirmation in larger studies in order to customize treatment with targeted therapies.

In this study, *PIK3CA, IRF2*, *ARID1A* and *HNF4α* genes harbored mainly indel mutations in the noncoding regions. According to previous studies, the mutation rate of *PIK3CA* in HCC is controversial, with absence of mutation cases detected in a study done in Japan whereas a high mutation rate of 35.6% was reported in studies done in Korea [14–16]. In the present study, we only detected high frequency of indel mutations in non-coding regions in *PIK3CA* gene. The discrepancies in rates of *PIK3CA* mutations are likely due to a number of factors including the specific exons that were sequenced, geographical variation and methods used for sample storage and DNA extraction. Thus, the importance of *PIK3CA* mutation and its implications in HCC tumorigenesis needs further investigations. In addition, we observed low indel mutations in *IRF2* and a relatively high mutation rate in *TP53* gene. Regarding mutations in the *IRF2, ARID1A* and *HNF4α* genes, our findings are in line with other reports which show low mutation rates in HBV-related HCC [41, 42], suggesting that these genes may not involve in HBV-related HCC tumorigenesis.

One limitation of this study is the small sample size. Therefore, future studies with a larger cohort which includes healthy control as well as HCC patients without HBV infection should be performed. With a larger cohort, analysis of the co-mutational profile of the six chosen genes together with other well-known HCC-related genes such as β-catenin would further delineate the role of these genes in HCC. Finally, to understand functional role of *FAT4* in HCC tumorigenesis, an in-depth analysis of gene expression in a larger cohort and using animal models could facilitate deeper perspectives on the biological significant of *FAT4* in HCC.

## Conclusions

This study identifies frequent genetic alterations in cancer-related genes especially *FAT4* illustrated the very high genomic complexity in HCC pathogenesis. The high frequency of non-synonymous mutations in *FAT4* implied its involvement in HCC development, though the biological significance of these genetic alterations is still unknown. A more comprehensive understanding of the functional effects of the genetic alterations on *FAT4* expression in the pathogenesis of HCC might prompt the development of more efficient therapeutic strategies as well as facilitate a better understanding of HCC tumorigenesis.

## Additional files

Additional file 1: Selected cancer-associated genes (DOCX 17 kb)

Additional file 2: Primer and siRNA information (DOCX 19 kb)

Additional file 3: Clinical characteristics of patients (DOCX 15 kb)

Additional file 4: Targeted sequencing quality metrics (DOCX 16 kb)

Additional file 5: Significant single nucleotide variants with functional consequences (DOCX 18 kb)

Additional file 6: Genes with synonymous mutations (DOCX 18 kb)

Additional file 7: List of genes with insertion and deletion (DOCX 19 kb)

Additional file 8: Verification of identified non-synonymous mutations (DOCX 286 kb)

## Data Availability

All the data obtained and materials analyzed in this research are available with the corresponding author upon reasonable request.

## Abbreviations

ALK: Anaplastic lymphoma kinase
ARID1A: AT-rich interaction domain 1A
BRAF: serine/threonine-protein kinase B-raf
BWA: Burrows-wheeler aligner
FAT4: FAT atypical cadherin 4
GATK: Genome analysis tool kit
HBV: Hepatitis B virus
HCC: Hepatocellular carcinoma
HCV: Hepatitis C virus
hg19: Human reference genome 19
HNF4α: Hepatocyte nuclear factor 4α
Indels: Insertions/deletions
IRF2: Interferon regulatory factor 2
LTR: Long-terminal repeat
NGS: Next generation sequencing
PIK3CA: Phosphatidylinositol 3-kinase subunit alpha
Polyphen: Polymorphism phenotyping
*SIFT*: Scale-invariant feature transform
TP53: Tumor protein p53
UTR: Untranslated region

## References

[1] Torre LA, Bray F, Siegel RL, Ferlay J, Lortet-Tieulent J, Jemal A (2015). Global cancer statistics, 2012. CA Cancer J Clin.

[2] Forner A, Llovet JM, Bruix J (2012). Hepatocellular carcinoma. Lancet.

[3] Altekruse SF, McGlynn KA, Reichman ME (2009). Hepatocellular carcinoma incidence, mortality, and survival trends in the United States from 1975 to 2005. J Clin Oncol.

[4] Llovet JM, Burroughs A, Bruix J (2003). Hepatocellular carcinoma. Lancet.

[5] Llovet Josep M., Montal Robert, Sia Daniela, Finn Richard S. (2018). Molecular therapies and precision medicine for hepatocellular carcinoma. Nature Reviews Clinical Oncology.

[6] Thorgeirsson SS, Grisham JW (2002). Molecular pathogenesis of human hepatocellular carcinoma. Nat Genet.

[7] Kwak EL, Bang YJ, Camidge DR, Shaw AT, Solomon B, Maki RG, Ou SH, Dezube BJ, Janne PA, Costa DB (2010). Anaplastic lymphoma kinase inhibition in non-small-cell lung cancer. N Engl J Med.

[8] Falchook GS, Long GV, Kurzrock R, Kim KB, Arkenau TH, Brown MP, Hamid O, Infante JR, Millward M, Pavlick AC (2012). Dabrafenib in patients with melanoma, untreated brain metastases, and other solid tumours: a phase 1 dose-escalation trial. Lancet.

[9] Villanueva A, Llovet JM (2014). Liver cancer in 2013: mutational landscape of HCC--the end of the beginning. Nat Rev Clin Oncol.

[10] Guichard C, Amaddeo G, Imbeaud S, Ladeiro Y, Pelletier L, Maad IB, Calderaro J, Bioulac-Sage P, Letexier M, Degos F (2012). Integrated analysis of somatic mutations and focal copy-number changes identifies key genes and pathways in hepatocellular carcinoma. Nat Genet.

[11] Cleary SP, Jeck WR, Zhao X, Chen K, Selitsky SR, Savich GL, Tan TX, Wu MC, Getz G, Lawrence MS (2013). Identification of driver genes in hepatocellular carcinoma by exome sequencing. Hepatology.

[12] Olivier M, Hollstein M, Hainaut P (2010). TP53 mutations in human cancers: origins, consequences, and clinical use. Cold Spring Harb Perspect Biol.

[13] Teufel A, Marquardt JU, Galle PR (2013). Next generation sequencing of HCC from European and Asian HCC cohorts. Back to p53 and Wnt/beta-catenin. J Hepatol.

[14] Lee JW, Soung YH, Kim SY, Lee HW, Park WS, Nam SW, Kim SH, Lee JY, Yoo NJ, Lee SH (2005). PIK3CA gene is frequently mutated in breast carcinomas and hepatocellular carcinomas. Oncogene.

[15] Colombino M, Sperlongano P, Izzo F, Tatangelo F, Botti G, Lombardi A, Accardo M, Tarantino L, Sordelli I, Agresti M (2012). BRAF and PIK3CA genes are somatically mutated in hepatocellular carcinoma among patients from South Italy. Cell Death Dis.

[16] Tanaka Y, Kanai F, Tada M, Asaoka Y, Guleng B, Jazag A, Ohta M, Ikenoue T, Tateishi K, Obi S (2006). Absence of PIK3CA hotspot mutations in hepatocellular carcinoma in Japanese patients. Oncogene.

[17] Schulze K, Nault JC, Villanueva A (2016). Genetic profiling of hepatocellular carcinoma using next-generation sequencing. J Hepatol.

[18] Zang ZJ, Cutcutache I, Poon SL, Zhang SL, McPherson JR, Tao J, Rajasegaran V, Heng HL, Deng N, Gan A (2012). Exome sequencing of gastric adenocarcinoma identifies recurrent somatic mutations in cell adhesion and chromatin remodeling genes. Nat Genet.

[19] Holbrook JD, Parker JS, Gallagher KT, Halsey WS, Hughes AM, Weigman VJ, Lebowitz PF, Kumar R (2011). Deep sequencing of gastric carcinoma reveals somatic mutations relevant to personalized medicine. J Transl Med.

[20] Samuels Y, Waldman T (2010). Oncogenic mutations of PIK3CA in human cancers. Curr Top Microbiol Immunol.

[21] Bolger AM, Lohse M, Usadel B (2014). Trimmomatic: a flexible trimmer for Illumina sequence data. Bioinformatics.

[22] Li H, Durbin R (2009). Fast and accurate short read alignment with burrows-wheeler transform. Bioinformatics.

[23] Van der Auwera GA, Carneiro MO, Hartl C, Poplin R, Del Angel G, Levy-Moonshine A, Jordan T, Shakir K, Roazen D, Thibault J *et al*: From FastQ data to high confidence variant calls: the Genome Analysis Toolkit best practices pipeline. *Curr Protoc Bioinformatics* 2013, 43:11 10 11–33. 10.1002/0471250953.bi1110s43 PMC4243306 25431634

[24] Kumar P, Henikoff S, Ng PC (2009). Predicting the effects of coding non-synonymous variants on protein function using the SIFT algorithm. Nat Protoc.

[25] Adzhubei IA, Schmidt S, Peshkin L, Ramensky VE, Gerasimova A, Bork P, Kondrashov AS, Sunyaev SR (2010). A method and server for predicting damaging missense mutations. Nat Methods.

[26] Schwarz JM, Rodelsperger C, Schuelke M, Seelow D (2010). MutationTaster evaluates disease-causing potential of sequence alterations. Nat Methods.

[27] Chun S, Fay JC (2009). Identification of deleterious mutations within three human genomes. Genome Res.

[28] Cadieux N, Lebel P, Brousseau R (1993). Use of a triplex polymerase chain reaction for the detection and differentiation of mycoplasma pneumoniae and mycoplasma genitalium in the presence of human DNA. J Gen Microbiol.

[29] Mahoney PA, Weber U, Onofrechuk P, Biessmann H, Bryant PJ, Goodman CS (1991). The fat tumor suppressor gene in Drosophila encodes a novel member of the cadherin gene superfamily. Cell.

[30] Gao YB, Chen ZL, Li JG, Hu XD, Shi XJ, Sun ZM, Zhang F, Zhao ZR, Li ZT, Liu ZY (2014). Genetic landscape of esophageal squamous cell carcinoma. Nat Genet.

[31] Shi JY, Xing Q, Duan M, Wang ZC, Yang LX, Zhao YJ, Wang XY, Liu Y, Deng M, Ding ZB (2016). Inferring the progression of multifocal liver cancer from spatial and temporal genomic heterogeneity. Oncotarget.

[32] Yu J, Wu WK, Li X, He J, Li XX, Ng SS, Yu C, Gao Z, Yang J, Li M (2015). Novel recurrently mutated genes and a prognostic mutation signature in colorectal cancer. Gut.

[33] Supek F, Minana B, Valcarcel J, Gabaldon T, Lehner B (2014). Synonymous mutations frequently act as driver mutations in human cancers. Cell.

[34] Sauna ZE, Kimchi-Sarfaty C (2011). Understanding the contribution of synonymous mutations to human disease. Nat Rev Genet.

[35] Qi C, Zhu YT, Hu L, Zhu YJ (2009). Identification of Fat4 as a candidate tumor suppressor gene in breast cancers. Int J Cancer.

[36] Villanueva A, Hoshida Y (2011). Depicting the role of TP53 in hepatocellular carcinoma progression. J Hepatol.

[37] Cho Y, Gorina S, Jeffrey PD, Pavletich NP (1994). Crystal structure of a p53 tumor suppressor-DNA complex: understanding tumorigenic mutations. Science.

[38] Hussain SP, Schwank J, Staib F, Wang XW, Harris CC (2007). TP53 mutations and hepatocellular carcinoma: insights into the etiology and pathogenesis of liver cancer. Oncogene.

[39] Kew MC (2003). Synergistic interaction between aflatoxin B1 and hepatitis B virus in hepatocarcinogenesis. Liver Int.

[40] Scorsone KA, Zhou YZ, Butel JS, Slagle BL (1992). p53 mutations cluster at codon 249 in hepatitis B virus-positive hepatocellular carcinomas from China. Cancer Res.

[41] Jhunjhunwala S, Jiang Z, Stawiski EW, Gnad F, Liu J, Mayba O, Du P, Diao J, Johnson S, Wong KF (2014). Diverse modes of genomic alteration in hepatocellular carcinoma. Genome Biol.

[42] Totoki Y, Tatsuno K, Yamamoto S, Arai Y, Hosoda F, Ishikawa S, Tsutsumi S, Sonoda K, Totsuka H, Shirakihara T (2011). High-resolution characterization of a hepatocellular carcinoma genome. Nat Genet.

