# Supplementary material for: Targeted genomic profiling identifies frequent deleterious mutations in FAT4 and TP53 genes in HBV-associated hepatocellular carcinoma
Source: BMC Cancer. 2019 Aug 8;19:789. doi: 10.1186/s12885-019-6002-9 (PMC6686555; doi:10.1186/s12885-019-6002-9)
Supplement: Supplementary file 1 — Selected cancer-associated genes (DOCX 17 kb) [file 12885_2019_6002_MOESM1_ESM.docx]

**Table S1.** Selected cancer-associated genes for targeted sequencing

| **Gene** | **Description** | **Functional properties** | **NCBI reference ID** | |
| --- | --- | --- | --- | --- |
|  |  |  | **mRNA** | **Protein** |
| ARID1A | AT-rich interaction domain 1A | Chromatin remodeling | NM_006015 | NP_006006.3 (2285aa) |
| IRF2 | Interferon regulatory factor 2 | Transcriptional activator | NM_002199 | NP_002190 (349aa) |
| HNF4α | Hepatocyte nuclear factor 4α | Transcription factor | NM_00457 | NP_000448  (474aa) |
| TP53 | Tumor protein p53 | Tumor suppressor | NM_001126115 | NP_000537.3  (393aa) |
| PIK3CA | Phosphatidylinositol 3-kinase subunit alpha | Oncogene | NM_006218 | NP_006209  (1068aa) |
| FAT4 | FAT atypical cadherin 4 | Putative tumor suppressor | NM_024582 | NP_078858.4  (4981aa) |
