# Supplementary material for: Targeted genomic profiling identifies frequent deleterious mutations in FAT4 and TP53 genes in HBV-associated hepatocellular carcinoma
Source: BMC Cancer. 2019 Aug 8;19:789. doi: 10.1186/s12885-019-6002-9 (PMC6686555; doi:10.1186/s12885-019-6002-9)
Supplement: Supplementary file 2 — Primer and siRNA information (DOCX 19 kb) [file 12885_2019_6002_MOESM2_ESM.docx]

**Table S2**: siRNA product information and primers used for Sanger sequencing and mRNA expression study

| **Target gene** | **Oligonucleotide sequence (5’-3’)** | **Genetic variant ID** | **Nucleotide change** | **Product size (bp)** |
| --- | --- | --- | --- | --- |
| FAT4-SV1-Forward | CCGAACCTGAGCCTAATCAA | rs6847454 | A>T | 198 |
| FAT4-SV1-Reverse | GTACACTTGCTGTGAAAAGA |  |  |  |
|  |  |  |  |  |
| FAT4-SV2-Forward | CATATCTGCTGGGGACAGGT | rs1039808 | C>T | 388 |
| FAT4-SV2-Reverse | CCACTGGCCACTACCTTCAG |  |  |  |
|  |  |  |  |  |
| FAT4-SV3-Forward | ATCAGCAGCCAATCTGACA | rs36052762 | C>G | 217 |
| FAT4-SV3-Reverse | ATTGTCCCTGAATCCACTGC |  |  |  |
|  |  |  |  |  |
| FAT4-SV4-Forward | CCCATGTTCCTGAAAATTCC | rs12508222 | G>A | 267 |
| FAT4-SV4-Reverse | CTTGGAGCATTGTCATTGATGT |  |  |  |
|  |  |  |  |  |
| FAT4-SV5-Forward | ATCGAGAAACCCTTCCCATC | rs1567047 | G>A | 492 |
| FAT4-SV5-Reverse | TGTGGCTTCACAGAGCCTAA | rs76491994 | A>C |  |
|  |  |  |  |  |
| FAT4-SV6-Forward | AAAGCCGTGAGAGTCTTCCA | rs12650153 | G>A | 242 |
| FAT4-SV6-Reverse | AGGACTCTGCAGGCACTCAT |  |  |  |
|  |  |  |  |  |
| FAT4-SV7-Forward | AGGCACTGCTGACAACACAC | rs1014867 | C>T | 339 |
| FAT4-SV7-Reverse | TTCCCTCCACCACTAGTCCA | rs17069858 | G>A |  |
|  |  |  |  |  |
| TP53-SV1-Forward | CAGACTTCCTGAAAACAACG | rs1042522 | G>C | 248 |
| TP53-SV1-Reverse | CAAGAAGCCCAGACGGAAAC |  |  |  |
|  |  |  |  |  |
| TP53-SV2-Forward | CAAGCAATGGATGATTTGAT | rs121912666 | T>G | 131 |
| TP53-SV2-Reverse | TCCGTCCCAGTAGATTACCA | rs28934571 | C>A |  |
| FAT4-mRNA-F | CACCCAATCAAGCACATCGAGA | - | - | 121 |
| FAT4-mRNA-R | TTTGAGATGGAGGCCAAGCTAT |  |  |  |
| TP53-mRNA-F | GTTCCGAGAGCTGAATGAG | - | - | 390 |
| TP53-mRNA-R | CCTTAAAATCTAAGCTGGTA | - | - |  |
|  |  |  |  |  |
| FAT4 siRNA (sense) | CCUUCAUUGUUGAUCGUUAtt | - | - | - |
|  |  |  |  |  |
| FAT4 siRNA (antisense) | UAACGAUCAACAAUGAAGGtg | - | - | - |
