# Supplementary material for: Targeted genomic profiling identifies frequent deleterious mutations in FAT4 and TP53 genes in HBV-associated hepatocellular carcinoma
Source: BMC Cancer. 2019 Aug 8;19:789. doi: 10.1186/s12885-019-6002-9 (PMC6686555; doi:10.1186/s12885-019-6002-9)
Supplement: Supplementary file 3 — Clinical characteristics of patients (DOCX 15 kb) [file 12885_2019_6002_MOESM3_ESM.docx]

**Table S3**. Clinical characteristics of patients with hepatocellular

carcinoma

| **Variable** | **Patients (n=8)** |
| --- | --- |
| Age, median (range), year | 55.5 (46-76) |
| Gender |  |
| Male | 7 |
| Female | 1 |
| Tumor size, median, (range), cm | 6.5 (2-12) |
| HBsAg |  |
| Positive | 8 |
| Negative | 0 |
| Tumor encapsulation |  |
| Yes | 4 |
| No | 4 |
| Venous invasion |  |
| Yes | 2 |
| No | 6 |
| Tumor differentiation |  |
| Well | 8 |
| Poor | 0 |
| TNM stage |  |
| Early stage I-II | 5 |
| Late stage III-IV | 3 |

HBsAg: hepatitis B surface antigen; TNM: tumor node metastasis
