# Supplementary material for: Targeted genomic profiling identifies frequent deleterious mutations in FAT4 and TP53 genes in HBV-associated hepatocellular carcinoma
Source: BMC Cancer. 2019 Aug 8;19:789. doi: 10.1186/s12885-019-6002-9 (PMC6686555; doi:10.1186/s12885-019-6002-9)
Supplement: Supplementary file 4 — Targeted sequencing quality metrics (DOCX 16 kb) [file 12885_2019_6002_MOESM4_ESM.docx]

**Table S4:** Targeted sequencing quality metrics of the 16 liver tissue samples

| **Sample ID** | **Number of reads**  **(After Filtering)** | **Number of reads mapped to human genome (hg19)** | **% of reads mapped to human genome (hg19)** | **Number of on target reads after duplicate reads removal** | **On target mean coverage** | **% coverage of target regions ( >=1X)** | **% coverage of target regions ( >=8X)** |
| --- | --- | --- | --- | --- | --- | --- | --- |
| D18N | 1,135,664 | 1,134,674 | 99.91% | 958,266 | 738 | 99.47% | 98.60% |
| D18T | 1,102,994 | 1,102,294 | 99.94% | 961,326 | 741 | 99.33% | 98.63% |
| D27N | 1,231,186 | 1,230,487 | 99.94% | 1,082,534 | 834 | 99.24% | 98.74% |
| D27T | 1,143,146 | 1,142,544 | 99.95% | 1,011,250 | 779 | 99.15% | 98.66% |
| D5N | 1,207,920 | 1,207,168 | 99.94% | 1,060,733 | 818 | 99.20% | 98.68% |
| D5T | 1,102,970 | 1,102,385 | 99.95% | 975,744 | 752 | 99.12% | 98.73% |
| D80N | 1,273,374 | 1,272,744 | 99.95% | 1,110,246 | 856 | 99.29% | 98.75% |
| D80T | 1,146,380 | 1,145,689 | 99.94% | 1,004,545 | 774 | 99.16% | 98.72% |
| D83N | 1,047,808 | 1,047,317 | 99.95% | 940,230 | 725 | 99.28% | 98.74% |
| D83T | 1,021,460 | 1,020,826 | 99.94% | 886,402 | 683 | 99.38% | 98.66% |
| D86N | 1,235,494 | 1,234,704 | 99.94% | 1,044,532 | 805 | 99.48% | 98.61% |
| D86T | 1,042,256 | 1,041,768 | 99.95% | 927,842 | 715 | 99.45% | 98.68% |
| D89N | 1,253,654 | 1,253,125 | 99.96% | 1,120,138 | 864 | 99.37% | 98.78% |
| D89T | 1,085,260 | 1,084,818 | 99.96% | 902,987 | 696 | 99.11% | 98.70% |
| D93N | 1,051,344 | 1,050,492 | 99.92% | 886,852 | 683 | 99.16% | 98.60% |
| D93T | 1,066,702 | 1,065,994 | 99.93% | 941,954 | 726 | 99.34% | 98.61% |
