# Supplementary material for: Targeted genomic profiling identifies frequent deleterious mutations in FAT4 and TP53 genes in HBV-associated hepatocellular carcinoma
Source: BMC Cancer. 2019 Aug 8;19:789. doi: 10.1186/s12885-019-6002-9 (PMC6686555; doi:10.1186/s12885-019-6002-9)
Supplement: Supplementary file 5 — Significant single nucleotide variants with functional consequences (DOCX 18 kb) [file 12885_2019_6002_MOESM5_ESM.docx]

**Table S5:** Significant single nucleotide variants with functional consequences in HBV-related HCC detected by targeted sequencing

| **Gene** | **SNP ID** | **Mutation type** | **Nucleotide**  **change** | **Amino acid change** | **PolyPhen 2**  **prediction** | **SIFT**  **prediction** | **LRT**  **prediction** | **Mutation**  **Taster prediction** |
| --- | --- | --- | --- | --- | --- | --- | --- | --- |
| FAT4 | rs6847454 | nonsynonymous | A1358T | Q453L | Benign | No prediction | Unknown | Possibly damaging |
| FAT4 | rs1039808 | nonsynonymous | C2420T | A807V | Possibly damaging | No prediction | Unknown | Possibly damaging |
| FAT4 | rs36052762 | nonsynonymous | C3769G | Q1257E | Possibly damaging | No prediction | Unknown | **Disease causing** |
| FAT4 | rs12508222 | nonsynonymous | G8476A | D2826N | Possibly damaging | Tolerated | Unknown | Possibly damaging |
| FAT4 | rs1567047 | nonsynonymous | G10571A | G3524D | **Probably damaging** | Tolerated | Unknown | Possibly damaging |
| FAT4 | rs76491994 | nonsynonymous | A10804C | I3602L | Possibly damaging | **Damaging** | Unknown | **Disease causing** |
| FAT4 | rs12650153 | nonsynonymous | G11618A | S3873N | Benign | Tolerated | Neutral | Polymorphism |
| FAT4 | rs1014867 | nonsynonymous | C14914T | P4972S | Benign | Tolerated | Unknown | Possibly damaging |
| FAT4 | rs17009858 | nonsynonymous | G14929A | A4977T | Benign | **Damaging** | Unknown | Polymorphism |
| TP53 | NA | nonsynonymous | C353G | P250R | **Probably damaging** | **Damaging** | **Deleterious** | **Disease causing** |
| TP53 | rs28934571 | nonsynonymous | G351T | R249S | **Probably damaging** | **Damaging** | **Deleterious** | **Disease causing** |
| TP53 | rs121912666 | nonsynonymous | A263C | Y220S | **Probably damaging** | **Damaging** | **Deleterious** | **Disease causing** |
| TP53 | rs1042522 | nonsynonymous | C98G | P72R | Benign | Tolerated | Unknown | Possibly damaging |

Note: SNP: single nucleotide polymorphism; NA: not available. Transcript ID: FAT4: NM_024582; TP53: NM_001126115.
