# Supplementary material for: Targeted genomic profiling identifies frequent deleterious mutations in FAT4 and TP53 genes in HBV-associated hepatocellular carcinoma
Source: BMC Cancer. 2019 Aug 8;19:789. doi: 10.1186/s12885-019-6002-9 (PMC6686555; doi:10.1186/s12885-019-6002-9)
Supplement: Supplementary file 6 — Genes with synonymous mutations (DOCX 18 kb) [file 12885_2019_6002_MOESM6_ESM.docx]

**Table S6:** Genes with synonymous mutations detected by targeted sequencing.

| **Gene Name** | **Chr** | **Position** | **SNP ID** | **Mutation type** | **Gene position** | **Nucleotide change** | **Amino acid**  **change** |
| --- | --- | --- | --- | --- | --- | --- | --- |
| FAT4 | Chr4 | 126237971 | rs72928772 | synonymous | exon | C405T | F135F |
| FAT4 | Chr4 | 126238397 | rs142490028 | synonymous | exon | G831A | A277A |
| FAT4 | Chr4 | 126240485 | rs144356360 | synonymous | exon | C2919T | V973V |
| FAT4 | Chr4 | 126240510 | rs2940779 | synonymous | exon | T2944C | L982L |
| FAT4 | Chr4 | 126241871 | rs2710555 | synonymous | exon | C4305T | I1435I |
| FAT4 | Chr4 | 126329789 | rs958415 | synonymous | exon | T5760C | D1920D |
| FAT4 | Chr4 | 126336703 | rs17009618 | synonymous | exon | T6585C | N2195N |
| FAT4 | Chr4 | 126369872 | rs988863 | synonymous | exon | G7701C | V2567V |
| FAT4 | Chr4 | 126371792 | rs148558216 | synonymous | exon | C9621T | A3207A |
| FAT4 | Chr4 | 126402890 | rs6824160 | synonymous | exon | G12813A | V4271V |
| FAT4 | Chr4 | 126412332 | rs1014865 | synonymous | exon | C14355T | V4785V |
| FAT4 | Chr4 | 126412575 | rs1014866 | synonymous | exon | A14598G | R4866R |
| IRF2 | Chr4 | 185310218 | rs1131553 | synonymous | exon | G744A | G248G |

Note: Chr: chromosome; SNP: single nucleotide polymorphism; Transcript ID: FAT4: NM_024582; IRF2: NM_002199.
