# Supplementary material for: Targeted genomic profiling identifies frequent deleterious mutations in FAT4 and TP53 genes in HBV-associated hepatocellular carcinoma
Source: BMC Cancer. 2019 Aug 8;19:789. doi: 10.1186/s12885-019-6002-9 (PMC6686555; doi:10.1186/s12885-019-6002-9)
Supplement: Supplementary file 7 — List of genes with insertion and deletion (DOCX 19 kb) [file 12885_2019_6002_MOESM7_ESM.docx]

**Table S7:** List of genes with insertion and deletion revealed by targeted sequencing

| **Gene Name** | **Chr** | **Position** | **SNP ID** | **Reference read** | **Alterative read** | **Variant type** | **Variant position** |
| --- | --- | --- | --- | --- | --- | --- | --- |
| ARID1A | Chr1 | 27092355 | NA | Ca | CAA | Indels | Intronic |
| ARID1A | Chr1 | 27107650 | NA | TA | T | Indels | UTR3 |
| PIK3CA | Chr3 | 178865774 | rs144390116 | G | GC | Indels | Upstream |
| PIK3CA | Chr3 | 178865958 | NA | AG | A | Indels | Upstream |
| PIK3CA | Chr3 | 178866053 | rs11367220 | GA | G | Indels | Upstream |
| PIK3CA | Chr3 | 178867958 | NA | CGTGT | CGTGTGT | Indels | Intronic |
| PIK3CA | Chr3 | 178917088 | rs10663584 | C | CATT | Indels | Intronic |
| PIK3CA | Chr3 | 178921253 | rs17550640 | G | GTAAA | Indels | Intronic |
| PIK3CA | Chr3 | 178927848 | NA | AT | ATTA | Indels | Intronic |
| PIK3CA | Chr3 | 178942267 | rs10605725 | CT | C | Indels | Intronic |
| PIK3CA | Chr3 | 178942269 | rs201266676 | ATAT | A | Indels | Intronic |
| PIK3CA | Chr3 | 178943987 | rs142501221 | T | TACTTG | Indels | Intronic |
| PIK3CA | Chr3 | 178952910 | NA | TA | T | Indels | Downstream |
| FAT4 | Chr4 | 126328633 | NA | C | CAA | Indels | Intronic |
| FAT4 | Chr4 | 126330259 | rs147990721 | AT | A | Indels | Intronic |
| FAT4 | Chr4 | 126354998 | rs143028724 | G | GCCTC | Indels | Intronic |
| FAT4 | Chr4 | 126390212 | NA | GT | G | Indels | Intronic |
| FAT4 | Chr4 | 126397808 | rs34205386 | TA | T | Indels | Intronic |
| FAT4 | Chr4 | 126398699 | NA | GT | G | Indels | Intronic |
| IRF2 | Chr4 | 185308975 | rs58025549 | TA | TAA,T | Indels | UTR3 |
| IRF2 | Chr4 | 185309360 | rs3832302 | CGGGATGGGATGGGAT | C,CGGGATGGGAT | Indels | UTR3 |
| IRF2 | Chr4 | 185394929 | NA | T | TCC | Indels | Intronic |
| TP53 | Chr17 | 7572154 | NA | GA | GAAA | Indels | UTR3 |
| TP53 | Chr17 | 7578711 | NA | CTTT | CT | Indels | UTR5 |
| TP53 | Chr17 | 7579643 | rs146534833 | CCCCCAGCCCTCCAGGT | C | Indels | Intronic |
| TP53 | Chr17 | 7580256 | NA | AT | A | Indels | Intronic |
| HNF4A | Chr20 | 43036397 | NA | TTGTG | TTGTGTG | Indels | Intronic |
| HNF4A | Chr20 | 43059237 | rs113151070 | TGAGGAAGAATGGTGTGGGA | T | Indels | UTR3 |
| HNF4A | Chr20 | 43060984 | NA | CTTTTTTTTTTT | C | Indels | UTR3 |
| HNF4A | Chr20 | 43061443 | rs141563916 | CA | C | Indels | UTR3 |
| HNF4A | Chr20 | 43061447 | NA | AG | A | Indels | UTR3 |

Note: Indel: insertion or deletions; UTR: untranslated region; Chr: chromosome; SNP: single nucleotide polymorphism; NA: not available
