# Supplementary material for: Targeted genomic profiling identifies frequent deleterious mutations in FAT4 and TP53 genes in HBV-associated hepatocellular carcinoma
Source: BMC Cancer. 2019 Aug 8;19:789. doi: 10.1186/s12885-019-6002-9 (PMC6686555; doi:10.1186/s12885-019-6002-9)

**Figure S1:** Verification of identified non-synonymous mutations predicted to have deleterious effect on protein function described in Additional file 5: Table S5 by Sanger sequencing method. Representative cases showing the sequencing electrophoretogram of genetic variants in *FAT4* (a-i) and *TP53* (a-c). Position of mutation is underlined and indicated with an arrow. The number is based on NCBI reference sequence for *FAT4* (NM_024582) and *TP53* (NM_001125115). N: non-tumor sample; T: tumor sample.


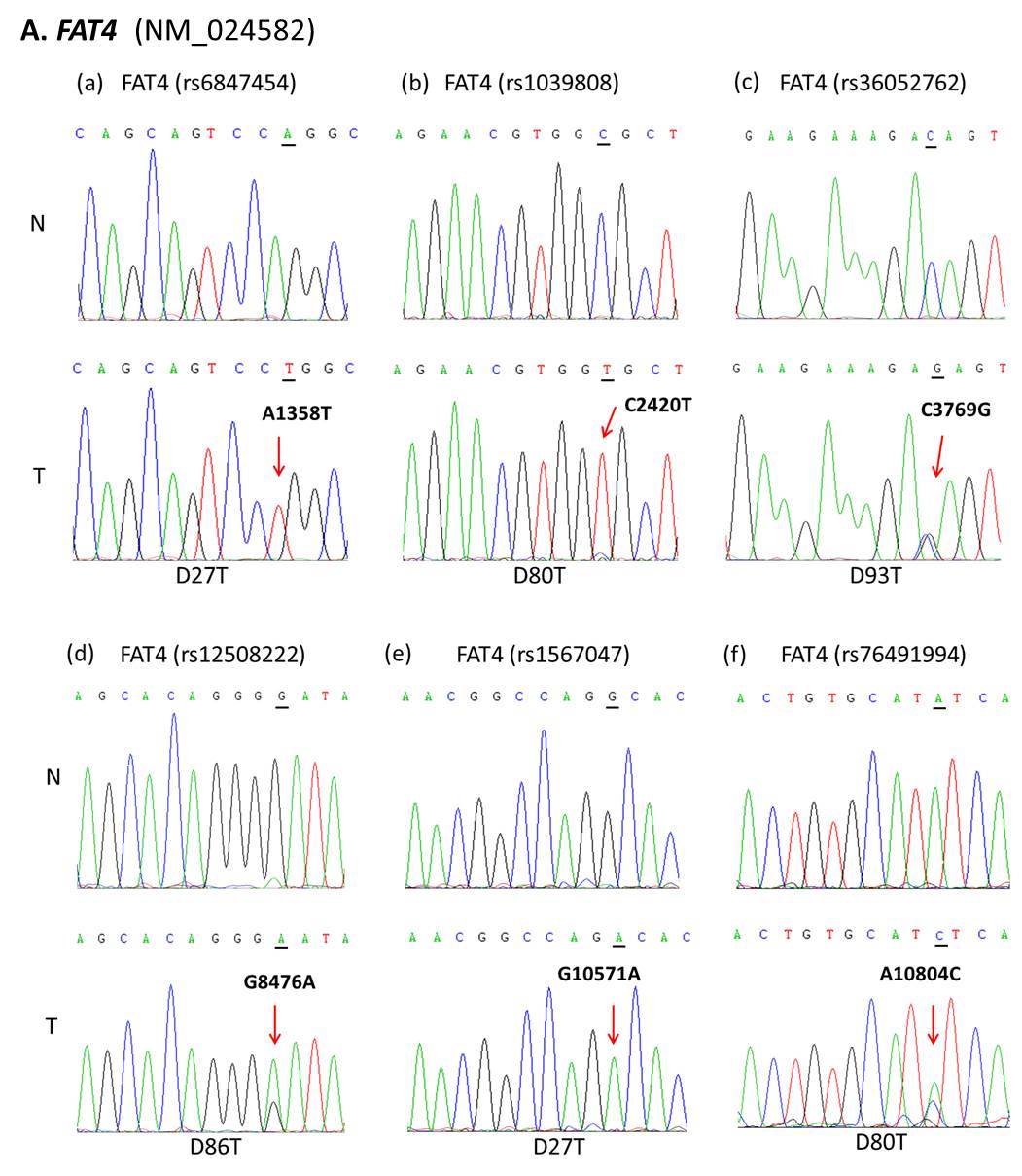


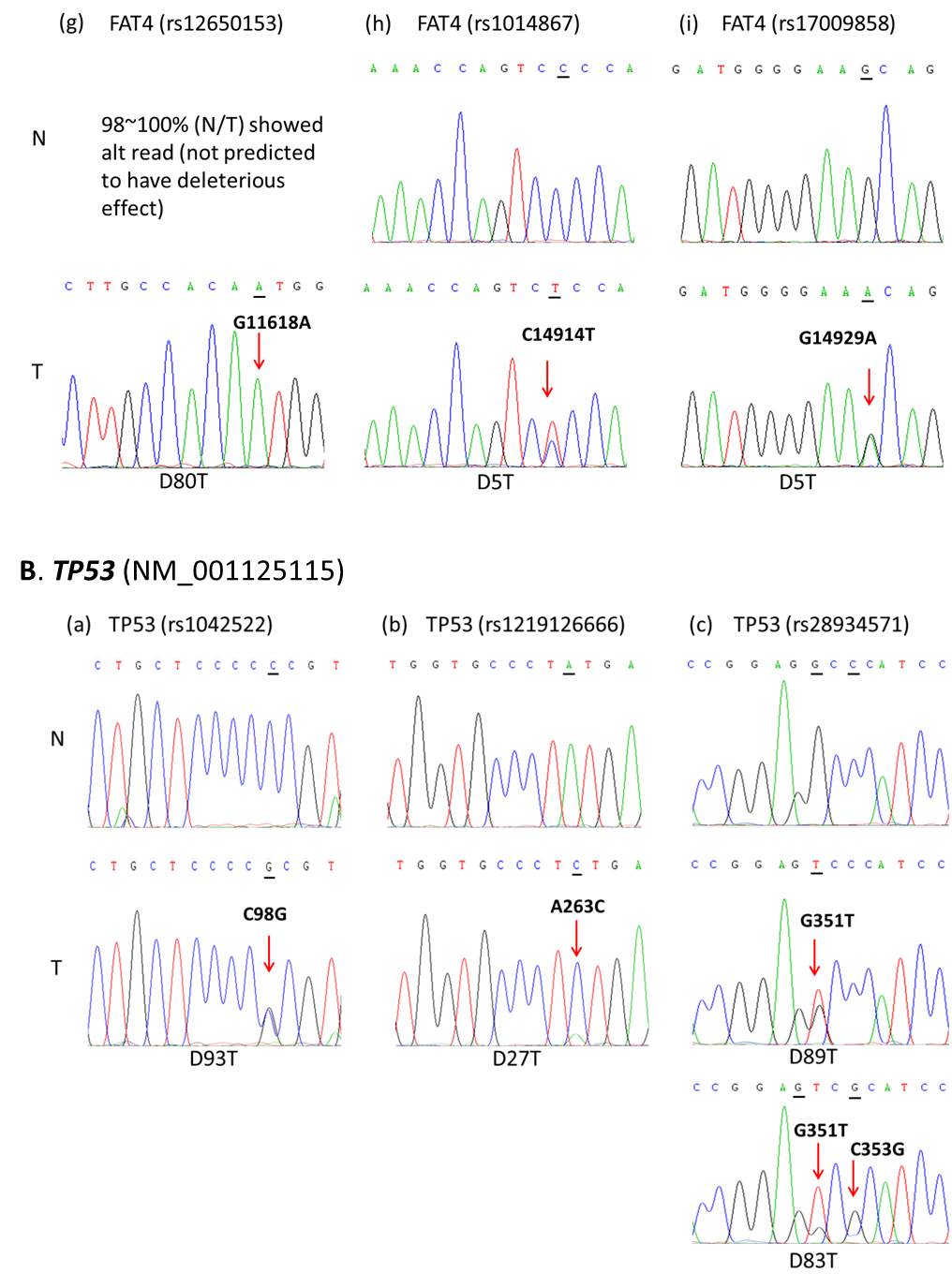

Supplement: Supplementary file 8 — Verification of identified non-synonymous mutations (DOCX 286 kb) [file 12885_2019_6002_MOESM8_ESM.docx]
